# Phospho-Chitooligosaccharides below 1 kDa Inhibit HIV-1 Entry In Vitro

**DOI:** 10.3390/cimb46040232

**Published:** 2024-04-22

**Authors:** Fatih Karadeniz, Se-Kwon Kim

**Affiliations:** 1 Marine Biotechnology Center for Pharmaceuticals and Foods, College of Medical and Life Sciences, Silla University, Busan 46958, Republic of Korea; 2 Department of Marine Science and Convergence Engineering, Hanyang University, ERICA Campus, 55 Hanyangdae-ro, Ansan 11558, Republic of Korea

**Keywords:** chitosan oligosaccharide, gp120, HIV-1, p24, phosphorylation

## Abstract

Despite present antiviral agents that can effectively work against HIV-1 replication, side effects and drug resistance have pushed researchers toward novel approaches. In this context, there is a continued focus on discovering new and more effective antiviral compounds, particularly those that have a natural origin. Polysaccharides are known for their numerous bioactivities, including inhibiting HIV-1 infection and replication. In the present study, phosphorylated chitosan oligosaccharides (PCOSs) were evaluated for their anti-HIV-1 potential in vitro. Treatment with PCOSs effectively protected cells from HIV-1-induced lytic effects and suppressed the production of HIV-1 p24 protein. In addition, results show that PCOSs lost their protective effect upon post-infection treatment. According to the results of ELISA, PCOSs notably disrupted the binding of HIV-1 gp120 protein to T cell surface receptor CD4, which is required for HIV-1 entry. Overall, the results point out that PCOSs might prevent HIV-1 infection at the entry stage, possibly via blocking the viral entry through disruption of virus–cell fusion. Nevertheless, the current results only present the potential of PCOSs, and further studies to elucidate its action mechanism in detail are needed to employ phosphorylation of COSs as a method to develop novel antiviral agents.

## 1. Introduction

For decades, natural origin molecules have been the center of attention as therapeutic agents or lead molecules for drug discovery due to their favorable properties, such as biodegradability, easier gastrointestinal absorption, and low cellular toxicity [1]. Polysaccharides are natural molecules that show therapeutic activities against several ailments, varying from tumor growth to viral infection [2,3]. Chitosan is a natural polysaccharide obtained through partial deacetylation of chitin, the second most abundant polysaccharide in nature following cellulose [4]. Both chitin and chitosan are regarded as essential lead molecules with biologically active chemical structures. Studies have reported beneficial effects on human health for both polysaccharides [5]. However, poor water solubility and low intestinal absorption hindered their utilization in functional food and pharmaceutical applications. In this context, chitosan oligosaccharides (COSs) were developed from enzymatic or chemical hydrolysis of chitosan, with properties such as water solubility, shorter chains, and monomers with free amino groups [6]. Reports already show that COSs have numerous bioactivities, such as antioxidant [7], antitumor [8], antibacterial [9], anti-obesity [10], anti-allergic [11], anti-diabetic [12], anti-osteoporotic [13], anti-inflammatory [14], and hepatoprotective [15] activities. Chemical modification of COSs also revealed that the biological effects of COSs could be varied and enhanced. To date, COSs have been modified via conjugation with lipids [16], phenolic acids [17], and amino acids [18], which enhanced their bioactivities. COSs have also been subjected to carboxylation [19], sulfation [20], and phosphorylation [21]. Compared to unmodified COSs, these derivations improved COS bioactivity in some cases, adding new bioactivities which COS itself did not possess [22].

Human Immunodeficiency Virus type 1 (HIV-1) is a retrovirus that causes acquired immunodeficiency syndrome (AIDS), a life-threatening condition that weakens the immune system and makes individuals vulnerable to opportunistic infections. Despite the availability of antiretroviral therapies that can effectively suppress HIV-1 replication, these drugs are not always well tolerated and can have significant side effects [23]. Therefore, there is a continued need to discover new and more effective HIV-1 inhibitors, particularly those derived from natural sources [24,25,26]. Studies focus on natural-origin HIV-1 inhibitors due to their more complex and varied chemical structures, low toxicity, and fewer side effects, which propose a more significant potential for inhibiting HIV-1. Natural products with anti-HIV-1 activities are also reported to possess broad antiretroviral potential [27]. Their ability to inhibit HIV-1 enzymes was postulated to translate into efficiency in inhibiting the same enzymes in other retroviruses. Also, polysaccharide antivirals such as dextran sulfate were reported to exert antiviral properties against the influenza, mumps, and rabies viruses. Although chitosan itself or chitosan conjugated with peptides and other classes of bioactive materials were mostly used in drug delivery or non-viral gene transfection approaches [28], studies showed that chitosan derivatization adding short peptide chains or amino acids are beneficial towards enhancing bioactivity as well. Lv et al. [29] showed that *N*-arginine-chitosan was able to enhance the antiviral efficiency of drug adefovir. Also, chitosan was studied as an adjuvant for antiviral vaccines to improve their efficiency [30,31]. Also, Artan et al. [32] reported that sulfation of low molecular COS exerted an in vitro anti-HIV-1 inhibitory effect by inhibiting entry stages. This article evaluated the anti-HIV-1 effect of phosphorylated COS (PCOS). Current findings suggest that PCOS holds promise as a lead molecule for developing a COS-based anti-HIV agent.

## 2. Materials and Methods

### 2.1. Materials and PCOS Synthesis

Chitosan oligosaccharides (below 1 kDa) were kindly donated by Kitto Life Co. (Seoul, Republic of Korea). Chemicals and reagents for the PCOS synthesis were purchased from Sigma Chemical Co. (St. Louis, MO, USA). All the other chemicals were of analytical grade and purchased from Junsei Chemical Co. (Tokyo, Japan). The reagent 3-(4,5-dimethylthiazol-2-yl)-2,5-diphenyltetrazolium bromide (MTT) and dimethyl sulfoxide (DMSO) were bought from Sigma Chemical Co. (St. Louis, MO, USA). The cell culture medium (RPMI 1640), penicillin/streptomycin, fetal bovine serum (FBS), and other cell culture materials were obtained from Gibco BRL, Life Technology (Grand Island, NY, USA). The H9 and H9/HIV-1_IIIB_ cell lines were obtained through the American Type of Culture Collection (Manassas, VA, USA). The C8166 cell line and HIV-1_RF_ strain from Dr. G. Farrar were provided by the EU Program EVA Center for AIDS Reagents, NIBSC, Potters Bar, UK.

PCOS was obtained and prepared according to the previously reported methods, with slight modifications [33,34]. The COSs used in PCOS synthesis were purchased and found to be deacetylated with degrees above 93%. Obtained PCOS samples were subjected to ^31^P NMR, the results of which suggest that the degree of phosphorylation was considerably high due to present phosphorylation at all reactive structural positions of COS: namely, the C_3_ and C_6_ OH groups along with the C_2_ amino group [33]. In the same study, it was also shown that the PCOS pyranose ring was stable under heat and acidic conditions, and the phosphorylated moiety removal was only observed at temperatures as high as 200 °C.

### 2.2. Cell Culture, HIV-1 Infection, Syncytia Formation, and Cell Viability Analysis

H9, H9/HIV-1_IIIB_, and C8166 cell lines were propagated at 37 °C under 5% CO_2_ in complete RPMI 1640 medium supplemented with 10% FBS, with 100 μg of streptomycin and 100 U of penicillin per ml. All cells were cultured in either T25 or T75 cell culture flasks. Cells were sub-cultured 2–3 times a week to yield a final concentration of 1 × 10^5^ cells/T-25 flask. HIV-1 infection of CD4+ human T cell C8166 cell lines is characterized by initial formation of large, multinucleated giant cells called syncytia, followed later by the destruction of these giant cells via swelling and death. Basically, 1 × 10^5^ cells/well C8166 cells in aliquots of 300 μL were seeded in triplicate to 48-well plates containing 100 μL of PCOS in different concentrations in complete RPMI-1640 medium with 5% FBS. After 2 h of incubation, the cells were infected with 100 μL of stock supernatant of HIV-1_RF_ diluted in complete medium at 200 CCID_50_ (μg/mL). The plates were incubated at 37 °C for 48 h, and the formation of syncytia was determined microscopically by using an inverted microscope (DMire2, Leica Microsystems, Wetzlar, Germany). The formed syncytia were counted.

To determine the protective effect of PCOSs against HIV-1-induced lysis of infected C8166 cells, an MTT-formazan-based assay was used. Cells in the log-growth phase were washed and resuspended in complete medium, and a 300 μL aliquot containing 1 × 10^5^ cells was added in triplicate to the wells of a 48-well plate containing different concentrations of compounds in a volume of 100 μL of medium. Stock supernatants of HIV-1_IIIB_ were diluted in complete medium to yield sufficient cytopathicity (~90% cell kill in 5 days) (CCID_50_), and a 100 μL aliquot was added to the wells. Plates were incubated for 5 days at 37 °C. After incubation, 100 μL of 500 μg/mL MTT solution was added to each well, and the plate was incubated for another 4 h at 37 °C. The formazan salt formed by viable cells was dissolved in equal parts of isopropyl alcohol (acidified with 40 nm HCl) and DMSO containing triton X-100 (4%). Optical density was measured at 540 nm with a GENios microplate reader (Tecan, Austria GmbH, Grödig, Austria). The optical density of formazan formed by untreated cells was taken as 100% viability, and the viabilities of treatment groups were given as percentages relative to this.

### 2.3. Measurement of p24 Antigen

To measure p24 antigen levels in HIV-1-infected cell culture medium, a commercial p24 antigen capture ELISA was used (Perkin-Elmer Life Sciences, Boston, MA, USA) following the manufacturer’s directions. Basically, H9 cells were cultured in 24-well plates at a density of 5 × 10^5^ cells/well and infected with HIV-1_RF_ as described in Section 3.2. Cells were treated with PCOS for five days, and the supernatants of culture wells were used to detect viruses released to the medium via p24 ELISA.

Expression of p24 protein was investigated via Western blotting in cellular fractions and culture medium. H9 cells were cultured and infected as described above. Cells were treated with PCOS for five days. To analyze the protein levels of p24, the total protein was isolated from cells through the addition of 1 mL of lysis buffer containing 50 mM of Tris-HCl, 0.4% (*w*/*v*) NP-40, 120 mM of NaCl, 1.5 mM of MgCl_2_, 2 mM of PMSF, 3 mM of NaF, and 1 mM of DTT to each well after harvesting the supernatant. Viral proteins were obtained from harvested supernatants, as described earlier [35]. On the other hand, cell lysates were centrifuged at 12,000 rpm for 10 min, and the supernatants were used for Western blotting. The protein concentration of samples was calculated using a BCA protein assay kit (Thermo Fisher Scientific, Waltham, MA, USA), and the twenty micrograms of protein from each well were loaded on a 10% SDS-PAGE gel. Following SDS-PAGE, proteins were transferred to nitrocellulose membranes. Blotted membranes were then blocked in 5% skim milk for 4 h at room temperature and hybridized with p24 monoclonal primary antibody overnight at 4 °C. Membranes were then subjected to horse radish peroxidase conjugated with anti-mouse antibody for 1 h at room temperature. Protein bands on membranes were visualized with a commercial chemiluminescence kit (Amersham ECL detection kit, GE Healthcare, Chicago, IL, USA), and the images were taken using the Fujifilm Imaging System (Fujifilm Life Science, Tokyo, Japan).

### 2.4. HIV-1 RT and Protease Activity Assay

The activity of HIV-1 reverse transcriptase, isolated from the virus pellet of H9/HIV-1_IIIB_ culture supernatant as described in earlier studies [11], was evaluated using a fluorescence RT assay kit (EnzChek, InvitroGen, Waltham, MA, USA) according to the manufacturer’s protocol. The activity of the HIV-RT was measured as the fluorescence intensity of the wells as a result of RT activity on the substrate. Fluorescence intensity was measured at 480 nm (excitation) and 520 nm (emission) with a GENios microplate reader (Tecan Austria GmbH, Grödig, Austria) after the addition of 173 μL of fluorescent PicoGreen reagent prepared in TE buffer.

In order to assess the protease inhibitory effect of the PCOS, the commercial SensoLyte 520 HIV-1 protease assay kit (Anaspec, CA, USA) was used according to the manufacturer’s directions. PCOSs were tested for their ability to inhibit proteolytic cleavage of the HiLyte Fluor™488/QXL™520 FRET peptide (obtained from the SensoLyte 520 HIV-1 protease assay kit) via HIV-1 protease. The amount of HiLyte Fluor™488 produced by successful protease activity was measured using the GENios^®^ microplate reader (Tecan Austria GmbH, Austria).

### 2.5. Post-Infection Treatment and Co-Culture Assays

Uninfected C8166 cells were cultured into individual wells of a 48-well microtiter plate at a density of 3 × 10^4^ cells/well in 400 μL of RPMI medium. Previously prepared diluted HIV-1_RF_ stock supernatants (100 μL) were added to appropriate wells to yield a final 200 CCID_50_ (μg/mL). At various times after the addition of the virus, a 100 μL aliquot of PCOS, saquinavir, and azidothymidine was added to multiple wells. Another set of cells was only treated with samples and incubated without being infected with the virus. After a total of 48 h of incubation, cellular viability was assessed using the MTT assay as previously described in Section 3.2, and protection ability was calculated in reference to treated yet uninfected cell groups.

For the co-culture assays, C8166 and H9 cells were cultured together. Briefly, C8166 cells were seeded in a 48-well plate (5 × 10^4^ cell/well) along with H9 cells (infected with HIV-1_IIIB_ as described in Section 3.2) in the ratio of 10:1 in the presence or absence of PCOS. The cells were incubated for 72 h, and the number of syncytia was counted using a fluorescent microscope, as noted in Section 3.2.

### 2.6. Analysis of gp120-CD4 Binding

The effect of PCOS on HIV-1 entry was investigated via its interaction of CD4-gp120 binding. ELISA was carried out as previously reported, with the required modifications [34]. Briefly, wells of an ELISA plate were coated with 2 μg/mL anti-gp120 antibody (Santa Cruz Biotechnology, Santa Cruz, CA, USA) in carbonate buffer (pH 9.6) and incubated overnight at 4 °C. Wells were then blocked with PBS containing 0.1% BSA at 37 °C for 1 h. Recombinant HIV-1_IIIB_ (100 ng/well) in PBS was added to each well, and the plate was incubated at 37 °C for 1 h. The wells were then washed with PBS containing 0.05% Tween 20. Different concentrations of PCOS were added to each well along with human sCD4 (100 ng/well), and the plates were incubated further at 37 °C for 1 h. After that, the incubation wells were treated with anti-sCD4 IgG (250 ng/mL), which was followed by another incubation at 37 °C for 1 h. Biotinylated IgG against previously treated anti-sCD4 IgG and subsequent horseradish peroxidase conjugated with streptavidin were added for the detection of gp120-bound CD4 proteins. Measurement was carried out by detecting absorbance values of wells at 405 nm after adding water-soluble horseradish peroxidase substrate that produces the yellow color (o-phenylenediamine dihydrochloride).

### 2.7. Statistical Analysis

The statistical significance of the experimental data was determined and expressed as a mean of three independent experiments ± standard deviation (SD). Differences between the means were analyzed using the analysis of variance (ANOVA) procedure of Statistical Analysis System, SAS v9.2 (SAS Institute, Cary, NC, USA), with Duncan’s multiple range test as a post-hoc analysis. The significance of differences was defined at the *p* < 0.05 level.

## 3. Results and Discussion

### 3.1. Anti-HIV Activity of PCOS

**Figure 1:**
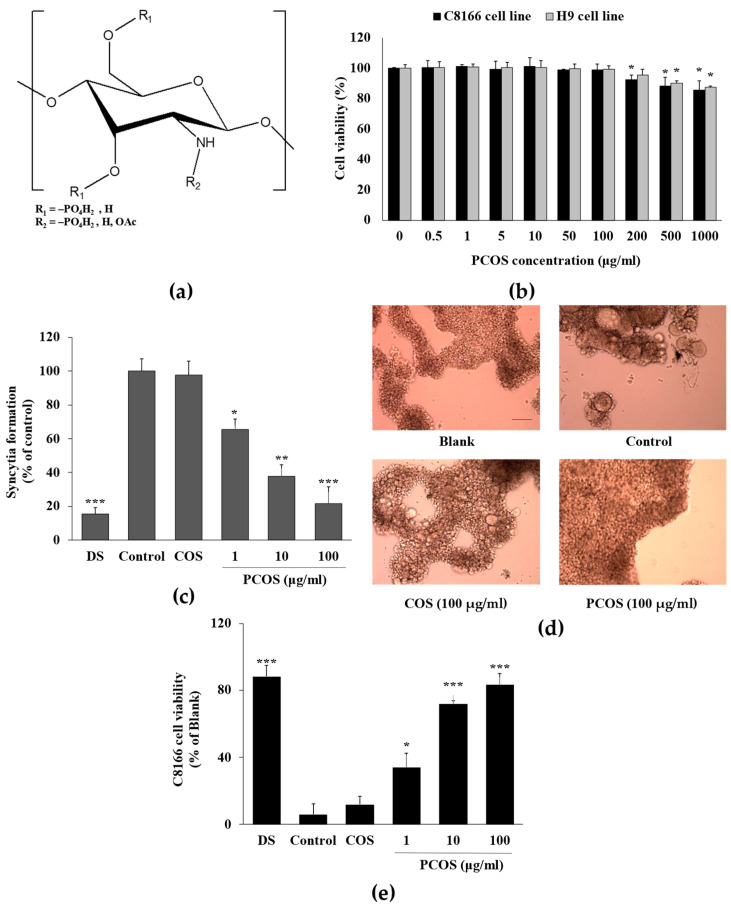
Effect of PCOSs on HIV-1-induced lytic effects in C8166 cells. (**a**) Chemical structure of PCOS. (**b**) Effect of PCOS treatment on cell viability of C8166 and H9 cell lines. Cells were treated with or without PCOSs at given concentrations and incubated for 5 days. Viability of cells was measured via MTT assay. * *p* < 0.05 vs. untreated (0 μg/mL) group. (**c**) Syncytia formation was counted on day 2 post-infection via optical microscope. Values are given as relative percentages of the HIV-1-infected untreated control. (**d**) Images of C8166 cells infected with or without HIV-1_RF_ on day 2 post-infection. Infected cells were treated with COSs or PCOSs at the concentration of 100 μg/mL. Blank: uninfected untreated cells; control: HIV-1-infected untreated cells. Scale bar: 50 μm. (**e**) Effect of PCOSs on HIV-1 induced lytic effect measured via cell viability. Viability of cells given as a relative percentage of the blank (uninfected untreated) group measured on day 5 post-infection. DS: dextran sulfate, 100 μg/mL. * *p* < 0.05, ** *p* < 0.01, and *** *p* < 0.001 vs. control (HIV-1-infected, untreated).

Initially, the anti-HIV-1 activity of PCOS (Figure 1a) was evaluated according to their ability to protect C8166 human T cells from HIV-1-induced lysis. In relation to HIV-1-induced lysis, the formation of giant multinucleated cell-like formations (syncytia) was also observed to confirm the ability of PCOS to inhibit HIV-1 infection. Prior to anti-HIV experiments, to avoid any cytotoxic presence of PCOS, which might appear as antiviral, the effects of PCOS on uninfected cells were tested. Results of the MTT assay show that PCOS did not show any cytotoxic presence for the C8166 cell line until 100 μg/mL, which was chosen as the highest dose in further experiments (Figure 1b). Starting from 200 μg/mL, PCOS presence exhibited a slight decrease in viable cell amount for this cell line. Although the first observed toxic dose for PCOS in the H9 cell line was at 500 μg/mL, to unify the results, H9 cell lines were also treated with 100 μg/mL PCOS as the highest concentration. The syncytia formation was observed in C8166 cells infected with X4 tropic HIV-1_RF_ to induce syncytia formation and consequent cell death due to lytic effects of HIV-1 infection. C8166 cells were imaged to confirm the syncytia formation and HIV-1 infection, and the syncytia formations were manually counted (Figure 1c,d). The inhibition of syncytia formation was evaluated as a relative percentage compared with the untreated infected cells. Results show that PCOS exhibited a significant dose-dependent inhibitory effect on syncytia formation (Figure 1c). At the concentration of 100 μg/mL, PCOS-treated cells formed 78.23% fewer syncytia. This ratio was 84.54% for dextran sulfate (DS), which was used as a positive control. DS was chosen as a positive control for its reported anti-HIV-1 activities [32,36], along with its similarity in chemical structure to PCOS, which might possess a similar action mechanism. The phosphorylated groups carry less acidic properties, which is considered the main antiviral action mechanism for sulfated polysaccharides compared to sulfation, which might explain the similar yet slightly lesser effectiveness of PCOS compared to DS. However, PCOS might affect the viral–host interaction not only via charge but also structural characteristics [37].

The treatment with PCOS also prevented C8166 cells from HIV-1-induced lytic effects according to the results of the MTT assay (Figure 1e). Without treatment, only 5.78% of HIV-1 infected C8166 cells were alive compared to uninfected cells. This number steadily increased with PCOS treatment to 83.24% at the 100 μg/mL concentration. These results suggest that PCOSs suppress HIV-1-induced syncytia formation and death in C8166 cells.

**Figure 2:**
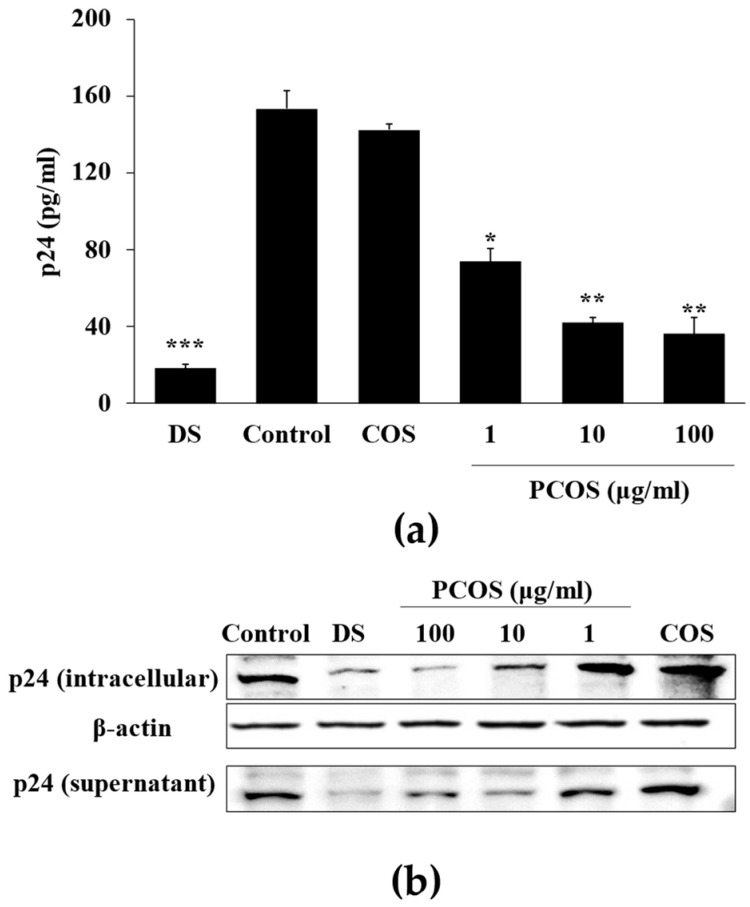
Effect of PCOS on HIV-1 p24 production in HIV-1_IIIB_-infected H9 cells. (**a**) p24 release in HIV-1 infected H9 cell culture medium was measured by p24 capture ELISA and given as pg per ml of supernatant. (**b**) Intracellular and supernatant p24 levels were detected by Western blot. DS: dextran sulfate, 100 μg/mL; Control: HIV-1 infected untreated cells. COS treatment was at the concentration of 100 μg/mL. * *p* < 0.05, ** *p* < 0.01 and *** *p* < 0.001 vs. Control (HIV-1 infected untreated).

Cytoprotective effects of PCOS against HIV-1-induced lysis were consistent with p24 ELISA and Western blotting results. Treatment with PCOS notably inhibited the p24 production in HIV-1 infected cells (Figure 2a). In addition to ELISA, Western blot analysis of intracellular and cell culture medium p24 protein levels was also analyzed. The production and release of the HIV-1 virus were related to the production of p24, as it is the most abundant protein of viral load [38]. Both intracellular and supernatant p24 levels of H9 cells infected with HIV-1_RF_ were suppressed after PCOS treatment (100 μg/mL) (Figure 2b). These results with the data obtained from syncytia formation and cytoprotective effect suggest that PCOS had a potential anti-HIV-1 effect, although they did not elucidate how this effect was exerted.

Overall, results were consistent with previous reports that showed phosphorylation of polysaccharides might enhance anti-HIV-1 activity. Feng et al. [39] synthesized phosphorylated derivatives of polysaccharides obtained from *Cyathula officinalis* and tested their antiviral potential. They concluded that phosphate graft quantity was directly linked with the antiviral effect of polysaccharides. The most effective antiviral polysaccharides were the ones with higher phosphorylation rates. The results suggest that the effect of phosphorylation provided the antiviral potential of isolated polysaccharides. In another study, Ming et al. [40] prepared a phosphorylated *Codonopsis pilosula* polysaccharide and demonstrated its superior antiviral effect on duck hepatitis A virus over pre-modification polysaccharides. They suggested that phosphorylation provided the *C. pilosula* polysaccharides with enhanced antiviral properties. Accordingly, the current results show that the anti-HIV-1 activity observed stemmed from phosphorylation. PCOSs showed significantly enhanced HIV-1 inhibitory effects compared to unmodified COSs.

### 3.2. Mechanism of Action

**Figure 3:**
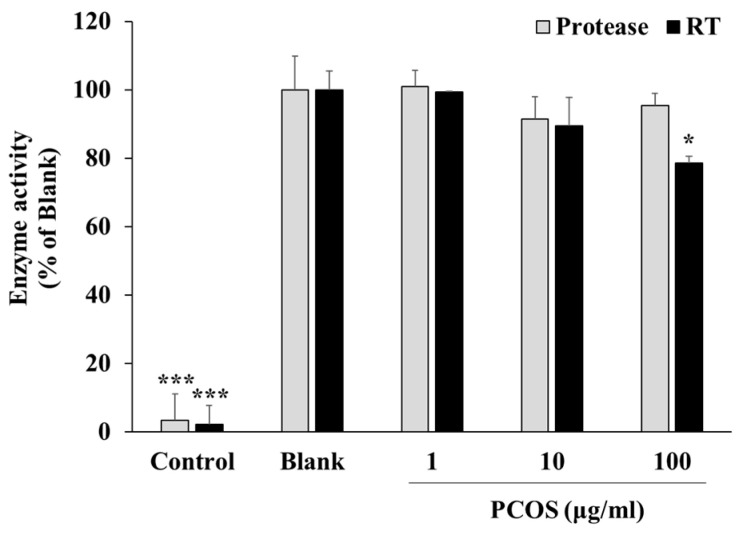
Effect of PCOSs on enzymatic activity of HIV-1 protease and RT. Enzymatic activities of HIV-1 RT and protease were measured with commercial assay kits as directed. Control: azidothymidine (AZT, 5 μM) and saquinavir (SQV, 5 μM) were used in the positive control as RT and protease inhibitors, respectively. Blank: HIV-1 RT or protease enzyme without any treatment. * *p* < 0.05, and *** *p* < 0.001 vs. blank.

Following confirmation of the anti-HIV-1 potential of PCOSs in vitro, further assays were performed to provide insights into the action mechanism of HIV-1 inhibition via PCOSs and to better understand at which stage of viral cycle PCOSs intervened. Primarily, the effect of PCOSs on the inhibition of HIV-1 reverse-transcriptase (RT) and protease was investigated. Drugs that act via inhibiting enzymes allocate a notable portion of orally administered drugs [41]. This was also the case for anti-HIV medication, where most of the approved HIV-1 drugs were inhibitors of HIV-1 enzymes, such as RT inhibitors that inhibit viral RNA replication, integrase inhibitors that prevent viral genome insertion, and protease inhibitors that stop the production of infectious viral particles. The protease activity assay revealed that PCOSs did not inhibit protease activity. No significant change was observed in protease activity after PCOS treatment (Figure 3), while treatment with saquinavir, a protease inhibitor, showed complete inhibition. Similar results were obtained with the HIV-RT activity assay for treatment with PCOS at 1, 10, and 100 μg/mL concentrations. At 100 μg/mL concentration, PCOS inhibited RT activity by 21.34%, which was not comparable to the complete inhibition via RT inhibitor azidothymidine treatment. IC_50_ for the RT inhibitory effect of PCOSs was 358.94 μg/mL. According to the results, it was suggested that PCOSs did not exert their anti-HIV-1 effect via enzyme inhibition.

**Figure 4:**
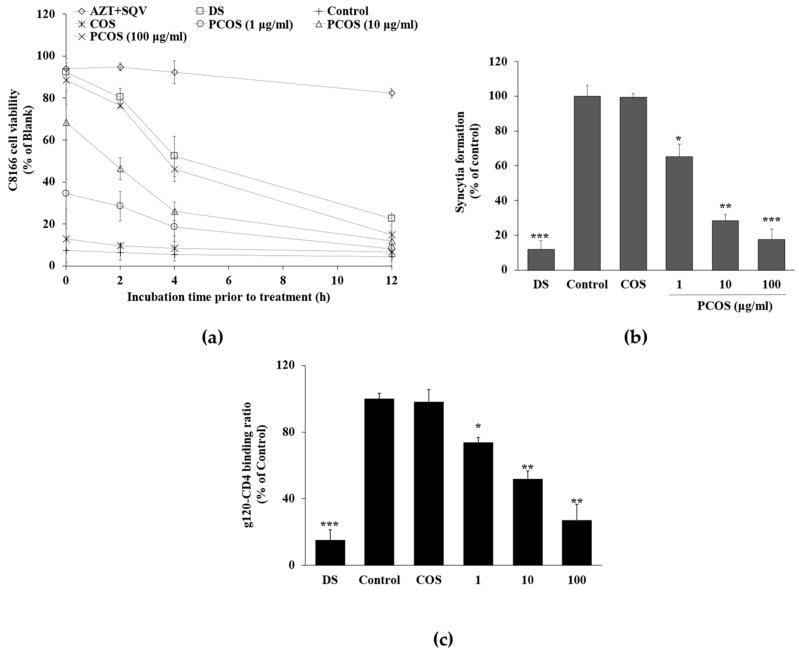
Effect of delayed addition of PCOSs on HIV-1-induced lytic effects (**a**), syncytia formation in co-culture (**b**), and HIV-1 gp120-CD4 interaction. (**a**) C8166 cells were infected with HIV-1_RF_ and incubated for 0, 2, 4, and 12 h before being treated with PCOS, AZT+SQV (azidothymidine, 5 μM; saquinavir, 5 μM), or DS (dextran sulfate, 100 μg/mL). The viability of cells was measured using MTT assay at day 5 post-infection. (**b**) Uninfected C8166 cells were co-cultured with H9 cells infected with HIV-1_IIIB_ at a ratio of 10:1 and treated with PCOS or DS (100 μg/mL). Syncytia formation was counted on day 5 post-infection and given as relative percentage of the control. Blank: uninfected untreated cells; control: HIV-1-infected untreated cells. (**c**) Effect of PCOSs on HIV-1 gp120 binding with cell receptor CD4 was investigated via ELISA. Binding was given as relative percentage of the control group, where gp120 and CD4 interaction did not interfere with any treatment. * *p* < 0.05, ** *p* < 0.01, and *** *p* < 0.001 vs. control.

To better understand at which stage PCOSs inhibited HIV-1, a post-infection treatment assay was carried out. PCOSs were introduced to the cells at certain times (0, 2, 4, and 12 h) after the cells were infected with HIV-1. Results show that the delayed addition of PCOS led to a decrease in viable cells on day five post-infection (Figure 4a). Starting from 2 h post-infection treatment, a time-dependent decrease was present in C8166 cell viability despite PCOS treatment. DS exhibited a similar pattern with PCOS, whereas the AZT-SQV cocktail maintained cell viability at 92.43% at day five post-infection despite being introduced to cells 4 h post-infection. However, event AZT-SQV treated cells showed a decrease in cell viability if the treatment was carried out 12 h post-infection. This result indicates that the effectiveness of PCOSs was dependent on whether they were present pre- or post-infection, further suggesting that PCOSs inhibited HIV-1 in the early stages of infection.

A co-culture assay was carried out to further confirm whether PCOSs might inhibit HIV-1 infection in the entry stage. Uninfected C8166 cells were co-cultured with HIV-1_IIIB_-infected H9 cells to observe the giant multinucleated cells, also called syncytia, formed as a result of interaction between infected and uninfected cells. The inhibitory activity of PCOSs on this interaction was investigated as an indicator for entry-stage inhibition. PCOSs effectively inhibited the cell–cell fusion, as 82.34% inhibited syncytia formation (Figure 4b). These data suggest that PCOSs could act as an entry inhibitor to exert their anti-HIV-1 effect. The entry stage of HIV-1 viral cycle starts with the binding of HIV-1 gp120 protein to a specific receptor on T cells called CD4 and its co-receptors. Therefore, the effect of PCOSs on the interaction between gp120 and CD4 was investigated with ELISA. Figure 4c showed that PCOS treatment dose-dependently hindered the binding of gp120 with CD4. Although the inhibitory effect of PCOSs at 100 μg/mL (72.78%) was lower than that of DS (84.61%), results indicate that the anti-HIV-1 effect of PCOSs might stem from the fact that they interrupt the HIV-1 entry stage.

Studies have reported that sulfated modification of polysaccharides enhanced their antiviral capabilities. Yoshida [42] postulated that the sulfate groups in sulfated polysaccharides might interact with positively charged amino acids in HIV-1 gp120. Similarly, Battulga et al. [36] reported the interaction between sulfated polysaccharides and HIV-1 surface protein gp120. Although phosphorylated modification of polysaccharides differs from sulfation, Zhou et al. [43] pointed out that both sulfated and phosphorylated modifications might alter the bioactivity mechanism because these modifications conduce the oxidant process of reducing carbon atoms inside the loops. Also, both modifications are known to increase solubility and therefore enhance the interaction between viral proteins and compounds. In this context, it was reported that HIV-1 envelope surface protein gp120 had binding domains with a specific affinity towards cell membrane-bound heparan sulfate [44]. This affinity between gp120 and sulfate groups of heparin sulfate was credited for the virus neutralization ability of antiviral agents with soluble polyanions. This interaction between gp120 and the cell membrane surface was found to occur through the V3 loop and was attributed to the anti-HIV-1 activities of sulfated polysaccharides [36,42]. There were similarities between phosphorylated and sulfated polysaccharide structures and charged side chains; the phosphorylated moieties of the polysaccharides were expected to interact with heparan sulfate binding domains. Reported interactions between sulfate groups and HIV-1 entry were consistent with the present results, which suggests that PCOS inhibited HIV-1 infection via interfering with virus–host cell binding, probably by preventing gp120-CD4 interaction.

## 4. Conclusions

In conclusion, the results show that PCOSs protected cells from HIV-1-induced death and reduced viral protein production. An interference at the early stages of HIV-1 infection, possibly disrupting the binding of viral gp120 to cell receptor CD4, was suggested to be the mechanism by which PCOSs exerted their anti-HIV-1 effect. Although the results of the current study suggest an HIV-1 entry inhibitory effect for PCOSs, future studies to elucidate their action mechanism in detail, especially the level of interaction between PCOSs and envelope proteins and cell surface receptors, are urged to utilize PCOSs as lead molecules for antiviral agent development. The current results further suggest that phosphorylation might strongly modulate the antiviral activity of polysaccharides, and hence, application of phosphorylation to other COS derivatives along with antiviral polysaccharides is expected to yield promising results towards discovery of novel natural antiviral compounds, especially against HIV-1 infection.

## Data Availability

Data used to support the findings of this study are available from the corresponding author upon reasonable request.
